# Development of an agent-based model to assess the impact of substandard and falsified anti-malarials: Uganda case study

**DOI:** 10.1186/s12936-018-2628-3

**Published:** 2019-01-09

**Authors:** Sachiko Ozawa, Daniel R. Evans, Colleen R. Higgins, Sarah K. Laing, Phyllis Awor

**Affiliations:** 1 Division of Practice Advancement and Clinical Education, UNC Eshelman School of Pharmacy, University of North Carolina, CB#7574, Beard Hall 115H, Chapel Hill, NC 27599 USA; 2 Department of Maternal and Child Health, UNC Gillings School of Global Public Health, University of North Carolina, Chapel Hill, NC USA; 3 Duke University School of Medicine, Durham, NC USA; 4 Department of Community Health and Behavioural Sciences, Makarere University School of Public Health, Kampala, Uganda

**Keywords:** Antimalarial, Quality, Substandard, Falsified, Agent-based model, Uganda

## Abstract

**Background:**

Global efforts to address the burden of malaria have stagnated in recent years with malaria cases beginning to rise. Substandard and falsified anti-malarial treatments contribute to this stagnation. Poor quality anti-malarials directly affect health outcomes by increasing malaria morbidity and mortality, as well as threaten the effectiveness of treatment by contributing to artemisinin resistance. Research to assess the scope and impact of poor quality anti-malarials is essential to raise awareness and allocate resources to improve the quality of treatment. A probabilistic agent-based model was developed to provide country-specific estimates of the health and economic impact of poor quality anti-malarials on paediatric malaria. This paper presents the methodology and case study of the Substandard and Falsified Antimalarial Research Impact (SAFARI) model developed and applied to Uganda.

**Results:**

The total annual economic impact of malaria in Ugandan children under age five was estimated at US$614 million. Among children who sought medical care, the total economic impact was estimated at $403 million, including $57.7 million in direct costs. Substandard and falsified anti-malarials were a significant contributor to this annual burden, accounting for $31 million (8% of care-seeking children) in total economic impact involving $5.2 million in direct costs. Further, 9% of malaria deaths relating to cases seeking treatment were attributable to poor quality anti-malarials. In the event of widespread artemisinin resistance in Uganda, we simulated a 12% yearly increase in costs associated with paediatric malaria cases that sought care, inflicting $48.5 million in additional economic impact annually.

**Conclusions:**

Improving the quality of treatment is essential to combat the burden of malaria and prevent the development of drug resistance. The SAFARI model provides country-specific estimates of the health and economic impact of substandard and falsified anti-malarials to inform governments, policy makers, donors and the malaria community about the threat posed by poor quality medicines. The model findings are useful to illustrate the significance of the issue and inform policy and interventions to improve medicinal quality.

**Electronic supplementary material:**

The online version of this article (10.1186/s12936-018-2628-3) contains supplementary material, which is available to authorized users.

## Background

Malaria is a preventable and treatable disease transmitted by infected mosquitoes. Since the millennium development goals listed combatting malaria as an important global development goal [1], significant progress has been made to combat malaria morbidity and mortality [2, 3]. Between 2006 and 2015, large-scale vector control campaigns, increased prophylactic treatments for pregnant women, as well as improved medical access, testing, and treatment methods reduced the global burden of malaria by an estimated 33 million cases and 435,000 deaths [2–4]. Unfortunately, this progress has stagnated and even regressed in recent years with malaria cases beginning to rise [2, 5].

This stagnation and regression in the battle against malaria represents a significant global health threat, especially in sub-Saharan Africa, where approximately 90% of the global malaria burden exists [2, 3, 5]. *Plasmodium falciparum* is a deadly species and disproportionally affects children under five, with the majority of malaria-related deaths occurring in this age group [6, 7]. In 2016, *P. falciparum* infections killed 285,000 children in Africa before they reached their fifth birthday [2, 7]. Malaria also poses a significant economic impact as a disease of poverty, and a cause of poverty [2, 8–11]. Malaria costs an estimated US$12 billion in direct costs to patients and their families annually, as well as hundreds of billions of dollars in indirect productivity losses [8]. These costs disproportionally affect low-and middle-income countries, instigating and reinforcing poverty rates and stunting national economic development [9–11].

One of the reasons for stalled progress in the fight against malaria can be traced to substandard and falsified (SF) anti-malarial treatments [12–15]. A recent meta-analysis estimates that approximately 19.1% of all anti-malarials in low- and middle-income countries are SF [16]. Substandard anti-malarials are defined as those that fail to meet the quality standards and/or specifications, while falsified anti-malarials deliberately and fraudulently misrepresent their identity, composition or source [17]. Anti-malarials as a medication class are the most likely to be falsified, and regional rates as high as 35% have been reported in Africa [13, 18]. Poor quality medicines directly affect health outcomes by increasing malaria morbidity and mortality, as well as threaten their effectiveness by contributing to artemisinin resistance [13–19, 20, 21]. SF anti-malarials also contribute to the economic burden of malaria through additional care-seeking and productivity losses from increased morbidity and mortality [19, 22].

Children under age five are at the greatest risk of malaria and also at the greatest risk to suffer from the consequences of SF anti-malarials [7, 13, 23]. The World Health Organization (WHO) estimates that SF anti-malarials in sub-Saharan Africa are responsible for 31,000–116,000 additional deaths, the majority of whom are children under five, resulting in US$10.4–38.5 million in avertable costs due to additional care-seeking [19]. While these estimates indicate the magnitude of the effect of poor quality medicines, robust, country-specific estimates are not available to combat the issue effectively and inform policy decisions [19]. This study developed the SAFARI (Substandard and Falsified Antimalarial Research Impact) model, an agent-based model simulation, to provide country-specific estimates of the health and economic impact of SF anti-malarials on paediatric malaria. This paper describes and presents model findings for Uganda, a country with particularly high under-five mortality due to malaria.

## Methods

The SAFARI model simulates population characteristics, malaria infection, patient care-seeking, disease progression, treatment outcomes, and associated costs, in order to estimate the health and economic impact of SF anti-malarial medicines among children below 5 years of age [19]. The model was developed using NetLogo software (Version 6.0.2). An agent-based model was utilized as it is adept at modelling complex adaptive systems [24]. The main model inputs and distributions are shown in Table 1 with additional inputs and coefficients included in Additional file 1. All model inputs were extracted from available literature and epidemiological outputs were compared to previously reported data. The primary outputs of the model are estimates of the health impact, direct costs, and productivity losses, based on the increased morbidity and mortality due to SF medicines. The model was presented to experts who helped validate the structure, inputs and assumptions.

**Table 1:** SAFARI model inputs

	Model inputs	Input	Range	Source
Demographic and epidemiological data	< 5 Population at risk	7,881,620		[25]
	Malaria incidence (annual rate for < 5 population at risk)	0.447	(0.197–0.744)	[26]
	Asymptomatic malaria case rate per 1000 population	0.156	(0.08–0.23)	[59]
	Probability that an untreated case progresses to a severe case	0.130	(0.07–0.3)	[60]
	Probability that a treatment failure progresses to a severe case	0.020	(0.005–0.05)	[61, 71]
	Case fatality rate for a severe case receiving quinine	0.109	(0.06–0.22)	[62]
	Case fatality rate for a severe case receiving other treatments	0.109	(0.06–0.22)	Assumption
	Case fatality rate for a severe case receiving ACTs	0.085	(0.06–0.22)	[62]
	Probability that a severe case progresses to NS	0.032	(0.028–0.035)	[62]
Healthcare-seeking behaviour	Care-seeking behaviour (%)			
	Public facilities	34.7%		[27]
	Private facilities	40.8%		
	Pharmacies	1.0%		
	Drug stores	5.6%		
	CHWs	0.7%		
	Self/neighbours	12.7%		
	No treatment	4.4%		
Medication stock by facility	Public facilities			
	% Stock ACTs	89.5%		[27]
	% Stock quinine	9.2%		
	% Stock other treatments^a^	1.3%		
	Private facilities			
	% Stock ACTs	77.2%		
	% Stock quinine	14.3%		
	% Stock other treatments	8.6%		
	Pharmacies			
	% Stock ACTs	76.0%		
	% Stock quinine	0.0%		
	% Stock other treatments	24.0%		
	Drug stores			
	% Stock ACTs	80.9%		
	% Stock quinine	19.1%		
	% Stock other treatments	0.0%		
	CHW2			
	% Stock ACTs	78.9%		
	% Stock quinine	0.0%		
	% Stock other treatments	21.1%		
	Self/neighbours			
	% Stock ACTs	87.2%		
	% Stock quinine	9.7%		
	% Stock other treatments	3.1%		
Probability facility has anti-malarial in stock	Public facilities	96.1%		[33]
	Private facilities	88.6%		
	Pharmacies	99.7%		
	Drug stores	86.1%		
	CHWs	99.7%		
	Self/neighbours (for ACTs)	100.0%		
Medication effectiveness	ACTs cure rate	0.9755	(0.9615–0.9895)	[35, 36, 38, 39, 41–49]
	Quinine cure rate	0.8818	(0.8484–0.9152)	[46, 49]
	Other treatments cure rate	0.7167	(0.6581–0.7753)	[37, 40, 47]
	No treatment cure rate	0		Assumption based on [32]
Proportions of SF medications	ACTs		Coefficient	
	Not SF (API > 85%)	80.5%	1	[51, 53, 56, 57]
	Category 1: API = 75–85%	10.5%	0.75	Adjusted [50, 54]
	Category 2: API = 50–75%	4.5%	0.5	
	Category 3: API < 50%	4.5%	0	
	Quinine			
	Not SF (API > 85%)	77.9%	1	[51–53, 55–57]
	Category 1: API = 75–85%	11.9%	0.75	Adjusted [50, 54]
	Category 2: API = 50–75%	5.1%	0.5	
	Category 3: API < 50%	5.1%	0	
	Other treatments			
	Not SF (API > 85%)	68.7%	1	[52, 55, 57]
	Category 1: API = 75–85%	16.9%	0.75	Adjusted [50, 54]
	Category 2: API = 50–75%	7.3%	0.5	
	Category 3: API < 50%	7.2%	0	
Treatment adherence	Number of doses taken		Coefficient	
	0–1	3.9%	0	[34]
	2	3.1%	0.25	
	3	7.3%	0.5	
	4	10.9%	0.75	
	5–6	74.7%	1	
Care-seeking behaviour	Number of days after onset of fever care was sought		Coefficient	[27]
	Same day	18.7%	0	
	1	32.0%	0.2	
	2	25.3%	0.4	
	3+	24.0%	0.6	

	Cost inputs^b^	Input	Range	Source
Patient out-of-pocket costs	Public facilities			
	Average cost of ACTs	$ 0.00		Assumption based on [27]
	Average cost of quinine	$ 0.00		
	Average cost of other treatments	$ 0.00		
	Private facilities			
	Average cost of ACTs	$ 2.59	(1.48–3.99)	[33]
	Average cost of quinine	$ 3.39	(2.75–4.08)	
	Average cost of other treatments	$ 0.65	(0.49–0.82)	
	Pharmacies			
	Average cost of ACTs	$ 2.91	(1.55–4.69)	
	Average cost of quinine	$ 2.72	(2.10–3.42)	
	Average cost of other treatments	$ 0.48	(0.32–0.66)	
	Drug stores			
	Average Cost of ACTs	$ 1.62	(1.05–2.31)	
	Average cost of quinine	$ 3.39	(2.76–4.08)	
	Average cost of other treatments	$ 0.48	(0.33–0.66)	
	CHWs			
	Average cost of ACTs	$ 0.00		Assumption based on [27]
	Average cost of quinine	$ 0.00		
	Average cost of other treatments	$ 0.00		
	Self/neighbours			
	Average cost of ACTs	$ 0.00		Assumption
	Average cost of quinine	$ 0.00		Assumption
	Average cost of other treatments	$ 0.00		Assumption
	Transport (pub, private)	$ 0.47	(0.39–0.55)	[64]
	Transport (pharmacy, drugstore)	$ 0.08	(0.04–0.12)	[65]
	Special foods for child	$ 1.15	(0.87–1.43)	[66]
	Supplemental medicines	$ 1.14	(1.02–1.26)	[64]
	Average testing costs	$ 0.91	(0.65–1.17)	[33]
	Private facility consultation costs	$ 4.35	(0–21.00)	[67]
	Cost per paediatric malaria hospitalization	$ 14.17	(0.75–47.50)	[67]
Productivity losses	Productivity loss per sick day	$ 1.59	(0.4–3.70)	[70]^c^
	Productivity losses from death	$ 14,959.66		[70]^d^
	NS disability productivity losses	$ 6189.87		[63, 70]^e^
Facility costs	Facility cost per testing	$ 1.46	(1.34–1.58)	[33, 64]
	Facility cost per ACTs	$ 1.50	(1.35–1.65)	[64]
	Facility cost for quinine	$ 2.48	(1.75–3.32)	[68]
	Facility cost per other treatments	$ 0.12	(0.03–0.26)	[68]
	Public facility cost per consultation	$ 8.58	(7.75–10.00)	[64]
	Facility cost per paediatric malaria hospitalization	$ 65.89	(59.55–72.45)	[69]
	Cost per CHW treatment	$ 1.17	(0.74–1.60)	[66]
	Cost per CHW testing	$ 1.09	(0.95–1.23)	[66]
	Facility cost per CHW visit	$ 4.63	(2.95–6.7)	[66]

*ACTs* artemisinin-based combination therapy, *API* active pharmaceutical ingredient, *CHW*s community health worker, *NS* neurological sequelae, *SF* substandard and falsified

^a^Other treatments included Sulfadoxine-pyrimethamine (SP), Chloroquine (CQ) and Amodiaquine (AQ)

^b^All costs are presented in US$2017

^c^ GDP per capita was divided by 365 days

^d^ GDP per capita was multiplied by disability-adjusted life expectancy

^e^ GDP per capita was multiplied by disability-adjusted life expectancy and disability weight

### Demographic characteristics, malaria infection and care-seeking rates

Utilizing agent-based modelling allows us to assign each child below age 5, or “agent-child” in the model, with individual demographic characteristics. Six demographic characteristics were applied to each agent-child: age, sex, geographic region, rural/urban, wealth quintile, and level of maternal education. These characteristics were chosen as important predictors that affect incidence, care-seeking, disease progression and treatment outcomes, and also to facilitate analysis by demographic groups. These characteristics were applied according to rates derived from the latest nationally representative Malaria Indicator Survey (MIS) using population sampling weights. For example, statistical analysis of the Uganda MIS dataset indicated significant associations between region, rural/urban, wealth quintile, and level of maternal education characteristics. To account for this association, the agent-child is sequentially assigned: (1) a region, (2) a rural/urban delineation according the regional proportions, (3) a wealth quintile according to the rural/urban proportions, and then (4) a maternal education level according to the proportions for the agent-child’s wealth quintile. Age in months (0–59) and sex were assigned randomly to each agent-child in the model. To ensure that the model population is comparable to a nationally representative sample [25], the proportions of each demographic characteristic in the model was calibrated to be within two percent of the MIS proportions.

In this model, each agent-child possesses individual incidence and care-seeking rates based on the demographic characteristics. Analysis of the Uganda MIS data showed significant associations between malaria prevalence and age, region, rural/urban, wealth quintile, and maternal education [27]. Further, significant associations were found between care-seeking and care sector seeking rates, and four of these characteristics besides age. The model accounts for these associations by deriving the prevalence and care-seeking coefficients for each demographic characteristic by dividing the categorical rate by the national rate. These coefficients are then multiplied by the incidence [26], care-seeking, and care sector seeking rates for each agent-child according to demographic characteristics. This allows the model to account for heterogeneity by giving each agent-child a unique individual incidence and care sector seeking rate. No significant association was observed between sex and malaria prevalence or between sex or age and care-seeking in Uganda. Beyond this Uganda example, associations between the six demographic characteristics can be analysed to determine which coefficients should be applied to the incidence and care/sector seeking rates in future SAFARI outputs.

### Disease progression

**Fig. 1:**
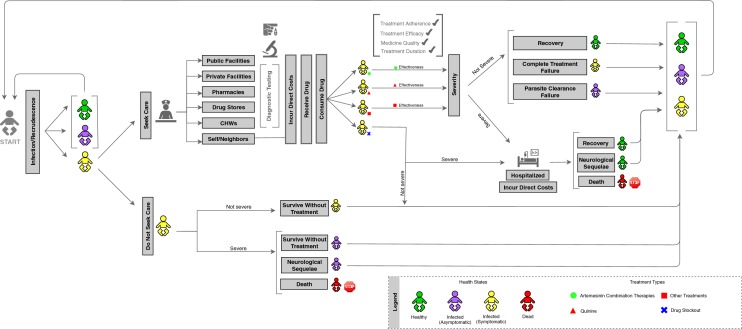
The SAFARI model flow diagram illustrates how the model simulates incidence, care-seeking, treatments, costs, and clinical outcomes

The disease model, shown in Fig. 1, is derived from the malaria disease progression established by the WHO, going from infection to asymptomatic parasitaemia, to uncomplicated illness, to severe malaria, and then to death [28]. The definitions for clinical outcomes and other components of the model are presented in Table 2 [29, 30]. The model simulates 1000 agent-children who are assigned individual characteristics, incidence, and care-seeking rates. A simulation was run over a 1-year period broken down into 5-day increments. The length of a period was set at 5 days to account for different rates of care-seeking and reported average duration of symptoms for a case of uncomplicated malaria receiving treatment [31]. There are four potential health states in the model: healthy, infected and asymptomatic, infected and symptomatic, and dead. Infected and symptomatic agent-children can develop neurological sequelae.

**Table 2:** Definitions utilized in the SAFARI model

Term	Definition
Malaria case	Occurrence of malaria infection in a person in whom the presence of malaria parasites in the blood can be confirmed by a diagnostic test [29]
Asymptomatic	“The presence of asexual parasites in the blood without symptoms of illness” [29]
Symptomatic	The presence of asexual parasites in the blood with symptoms of illness [29]
Recrudescence	“Malaria case attributed to the recurrence of asexual parasitaemia after anti-malarial treatment, due to incomplete clearance of asexual parasitaemia of the same genotype(s) that caused the original illness” [29]
Uncomplicated malaria	“Symptomatic malaria parasitaemia without signs of severity or evidence of vital organ dysfunction” [29]
Severe malaria	“Acute falciparum malaria with signs of severe illness and/or evidence of vital organ dysfunction” [29]
Treatment failure	“Inability to clear malarial parasitaemia or prevent recrudescence after administration of an anti-malarial medicine, regardless of whether clinical symptoms are resolved” [29]
Early treatment failure	Development of severe malaria or increase in parasitaemia during first three days of treatment or the presence of parasitaemia and a fever on third day of treatment [30]
Late clinical failure	Development of severe malaria or presence of parasitaemia and fever after three or more days since treatment began in cases that did not meet criteria for early treatment failure [30]
Complete treatment Failure/clinical failure	Complete treatment failure or clinical failure is equal to the sum of *Early Treatment Failure* and *Late Clinical Failure*” [30]
Parasite clearance failure	Presence of parasitaemia with no fever 1 week or longer after treatment began, also known as late parasitological failure [30]
Recovery	Recovery comes with adequate clinical and parasitological response, defined by absence of parasitaemia after 2 weeks indicating the elimination of all malaria parasites that caused the infection [29, 30]
Neurological sequelae	Deficits in cognition, gross motor function, speech, vision and hearing, behaviour problems or epilepsy resulting from severe malaria [28]
Drug efficacy	“Capacity of an anti-malarial medicine to achieve the therapeutic objective when administered at a recommended dose, which is well tolerated and has minimal toxicity” [29]
Treatment adherence	“Compliance with a regimen (chemoprophylaxis or treatment) or with procedures and practices prescribed by a health care worker” [29]
Substandard medicine	“Authorized medical products that fail to meet either their quality standards or specifications, or both” [18]
Falsified medicine	“Medical products that deliberately/fraudulently misrepresent their identity, composition or source” [18]

For each period, the 5-day incidence rate determines whether the agent-child will become infected and enter the disease model, or remain healthy. If the agent-child becomes infected, the agent-child can either stay asymptomatic, or become symptomatic and enter the care-seeking portion of the model. Symptomatic agent-children from the previous period that did not experience an adequate clinical and parasitogical response remain infected and symptomatic or become asymptomatic. Agent-children that are asymptomatic from the previous period will either become symptomatic or remain asymptomatic. Due to the longevity of *Plasmodium falciparum* infections, it is assumed that spontaneous recovery from malaria is not possible [32].

All symptomatic agent-children undergo simulated care-seeking and will either seek care or not, based on individual care-seeking rates. For agent-children seeking care, individual rates also determine the specific care location in which they will seek care. Analysis of MIS data determined the care locations incorporated in the model. For example, the Uganda model contains six care locations: public health facilities, private health facilities, pharmacies, drug stores, community health workers, and self-treatment/neighbours. The type of treatment the agent-child receives in each care location is determined by each location’s MIS-derived ratio of utilization of three anti-malarial treatments: artemisinin-based combination therapies (ACTs), oral quinine, and other treatments. Each care location also has a probability of stocking out of anti-malarial treatments based on ACTwatch data [33]. In the event that agent-children visit a care location during a stock out, they will receive no treatment and non-severe cases will remain symptomatic in the next period.

### Anti-malarial treatment outcomes

The treatment outcome for each agent-child was determined based on treatment adherence rates, treatment efficacy by medication, medicine quality measured by the active pharmaceutical ingredient (API) concentration of the specific drug the agent-child received, and treatment duration. Treatment adherence rates were derived from a study reporting the proportion of total malaria treatment taken, categorized by the numbers of doses taken: 0–1, 2, 3, 4, and 5–6 [34]. Treatment efficacy was modelled based on the doses taken, where lower adherence reduced the likelihood of successful treatment. If an agent-child pursued self-treatment or received medication from neighbours, the likelihood of treatment success was reduced by 20% due to lower adherence. Malaria treatment adherence was assumed to be similar throughout sub-Saharan Africa due to the lack of country-specific data available.

Treatment efficacy [35–49] and prevalence of SF medicines [50–57] for each anti-malarial type was calculated via the WWARN (Worldwide Antimalarial Resistance Network) database [58] and a systematic literature search specific to Uganda. The input for treatment efficacy was medication-specific likelihood of adequate clinical and parasitological response at 28 days based on polymerase chain reaction, controlling for recrudescence and reinfection. Treatment efficacy was estimated across studies by taking the weighted average of the success rate based on the number of people in the studies. The 95% confidence interval for treatment efficacy was derived based on the standard deviations of included studies. While SF medicines have API concentrations outside of the therapeutic range, model inputs acknowledge that not all are ineffective. Medication effectiveness was assumed to decrease proportionally by API concentration [19, 23], categorized according to API percentages between 75 and 85%, 50 and 75%, and < 50%, as interpolated from two studies [50, 54]. The prevalence of SF medicines was estimated by counting SF samples identified over all samples tested across studies. These SF medicines were then categorized based on the likelihood of having percentage API levels. Each modelled anti-malarial medication was assigned an API percentage category and corresponding treatment efficacy with lower APIs reducing the likelihood of successful treatment. The proportion of SF medicines for each anti-malarial category was assumed to be equivalent across care sectors, as no other data were available to segment medication quality by treatment location.

This model differentiates between symptomatic recovery and complete recovery, resulting in three outcomes for non-severe malaria in the model: recovery, complete treatment failure, and parasite clearance failure. Agent-children who experience treatment failure due to anti-malarials with an API < 50% or not adhering to treatment (taking only 0–1 doses) experience complete treatment failure and remain symptomatic. All other agent-children that experience treatment failure are considered to experience parasite clearance failure and remain infected but asymptomatic [59].

### Severe malaria

The probability of untreated symptomatic malaria progressing to severe malaria increases over time [28]. This is accounted for by using a severity rate that captures the country-specific rate of progression of an untreated uncomplicated case to a severe case, as reported in a Delphi study based on the country’s endemicity [60]. This severity rate is adjusted for each agent-child seeking care according to the amount of time from onset of symptoms until treatment. To determine these rates, MIS data were used to assign each agent-child with the number of days after onset of symptoms that care was sought, effectively capturing treatment duration. The severity rate for agent-children that seek care was adjusted to increase proportional to the number of days since onset [61]. The severity rate was not adjusted for agent-children that do not seek care.

In this model, agent-children that seek care with severe malaria become hospitalized and receive inpatient care, while those that do not initially seek care have a chance to seek care again in the next cycle. Inpatient hospital treatments were assumed to be of acceptable quality and patients admitted to hospitals were assumed to adhere to treatment.

There are four possible severe malaria outcomes in the model: death, neurological sequelae, remaining infected, and treatment success. Patients who receive inpatient care can be successfully treated, die, or experience neurological sequelae based on proportions reported by the AQUAMAT study in Africa [62]. Patients who do not receive care with severe malaria can remain infected, suffer from neurological sequelae, or die. A Delphi study estimated the mortality rate for severe cases that do not receive treatment [60].

The model tabulates the number of cases, neurological sequelae, and deaths due to malaria. Disability-adjusted life years (DALYs) due to malaria was also estimated as a measure of overall disease burden expressed as the number of years lost due to ill-health, disability or early death. Non-age weighted and non-discounted DALYs using disability weights from the Global Burden of Disease study are presented [63].

### Economic outcomes

Economic outcomes in the model were categorized as either direct costs or indirect costs. Direct costs included costs for transportation, testing, drugs, consultation, and hospital costs [64–67]. Direct costs were further separated into those that were paid out-of-pocket versus those incurred by public facilities [68, 69]. Indirect costs included productivity losses incurred by care-takers, as well as productivity losses due to disability and death due to malaria. Productivity losses were estimated using the human capital approach, based on gross domestic product (GDP) per capita and duration of lost productivity [70]. Caregiver productivity losses were estimated for 5 days of lost earnings for a case of uncomplicated severe malaria. Lifetime productivity losses were estimated based on lost economic productivity between age 15 and life expectancy, discounted at 3%. Disability productivity losses were calculated by applying the disability weights for neurological sequelae to discounted lifetime productivity losses [63]. All costs are rounded to the nearest thousands and expressed in 2017 US dollars.

### Scenario analyses

Three scenarios are presented for this analysis: the baseline scenario, a second scenario with no SF anti-malarials, and a third scenario with emergence of antimicrobial resistance. For the scenario with no SF medicines, all medications were assumed to have an API > 85%. For the antimicrobial resistance scenario, treatment efficacies for ACT and quinine were assumed to be the same as those for other treatments. The health and economic impact of SF anti-malarials and artemisinin resistance were calculated by examining the difference between the baseline scenario and the other two scenarios. To assess if there was a significant difference between the baseline and scenario outputs, a two-sample t-test was used for epidemiological outputs, and a Wilcoxon ranked-sum test was used for cost outputs. All data analysis was conducted using Stata 14.2.

To account for the natural variation in epidemiological and cost inputs, each input was simulated to vary probabilistically, where inputs were randomly generated within a specific distribution. Epidemiological inputs were normally distributed and cost inputs came from a gamma distribution. The overall results demonstrate the best estimate and standard deviation based on variations across all inputs.

## Results

This case study presents the SAFARI model results for Uganda. At current incidence rates and population levels, the model estimates that there are around 3.5 million annual cases of malaria in Ugandan children under age five. Among the total population, we estimated nearly 11,000 cases of neurological sequelae and around 29,000 deaths, which contributed to approximately 2 million DALYs lost. For cases that pursued some medical treatment (94%), an average of 177,000 (5%) were hospitalized in a year, with nearly 13,000 (0.4%) resulting in death and 5700 (0.2%) suffering from neurological sequelae. These cases of malaria, neurological sequelae, and death resulted in almost 1.1 million DALYs lost annually, including 953,000 years of life lost to early death from malaria. These health outcomes contributed to a total estimated economic impact of $614 million. Those that sought medical treatment made up $403 million of the total impact, with approximately 66% of the total. Direct costs associated with care seeking was estimated at $57.7 million (14%) and caregiver productivity losses were projected at $141 million (35%). Simulated outcomes for total malaria burden and cases who sought care are presented in Table 3.

**Table 3:** The annual health and economic impact of malaria, substandard and falsified anti-malarials, and antimicrobial resistance, among children < 5 in Uganda

	Annual burden of malaria^a^		Annual burden of malaria among cases that sought treatment^b^		Impact of substandard and falsified anti-malarials^d^			Impact of antimicrobial resistance^e^		
	Baseline	95% CI	Baseline	95% CI	Impact	% Diff^c^	p-value	Potential Impact	% Diff^c^	p-value
Avg. # cases	3,528,304	(3,527,862–3528,747)	3,331,214	(3,330,797–3,331,632)	154	0%	0.62	− 841	0%	0.01*
Avg. # hospitalized	176,744	(175,095–178,393)	176,744	(175,095–178,393)	13,919	8%	< 0.01*	10,418	6%	< 0.01*
Avg. # with NS	10,924	(10,720–11,128)	5665	(5531–5798)	307	5%	0.11	259	5%	< 0.01*
Avg. # deaths	29,481	(29,089–29,874)	12,893	(12,668–13,117)	1121	9%	< 0.01*	884	7%	< 0.01*
DALYs	1,999,460	(1,968,489–2,030,430)	1,091,211	(1,074,943–1,107,478)	78,565	7%	< 0.01*	63,040	6%	< 0.01*
YLLs	1,733,368	(1,704,149–1,762,587)	953,001	(937,692–968,310)	71,113	7%	< 0.01*	56,681	6%	< 0.01*
Economic impact	$613,510,000	(605,555,098–621,465,371)	$402,945,000	(394,989,859–410,900,132)	$31,009,000	8%	< 0.01*	$48,455,000	12%	< 0.01*
Direct costs	$57,669,000	(57,550,551–57,787,308)	$57,669,000	(57,550,551–57,787,308)	$5,151,000	9%	< 0.01*	$15,539,000	27%	< 0.01*
Facility costs	$32,550,000	(32,469,063–32,631,682)	$32,550,000	(32,469,063–32,631,682)	$2,857,000	9%	< 0.01*	$8,013,000	25%	< 0.01*
Out-of-pocket costs	$25,119,000	(25,081,489–25,155,626)	$25,119,000	(25,081,489–25,155,626)	$2,294,000	9%	< 0.01*	$7,526,000	30%	< 0.01*
Productivity losses	$555,841,000	(547,706,922–563,975,688)	$345,276,000	(340,893,439–349,658,692)	$25,859,000	7%	< 0.01*	$32,916,000	10%	< 0.01*
Short-term	$88,273,000	(86,720,283–89,825,161)	$140,798,000	(129,970,519–151,626,292)	$8,083,000	6%	< 0.01*	$18,903,000	13%	< 0.01*
Disability	$47,952,000	(46,753,481–49,150,506)	$37,256,000	(36,329,319–38,182,459)	$2,019,000	5%	< 0.01*	$1,725,000	6%	< 0.01*
Death	$419,617,000	(411,128,280–428,104,899)	$167,222,000	(161,703,081–172,740,461)	$15,756,000	9%	< 0.01*	$12,287,000	7%	< 0.01*

*Avg #* average number, *CI* confidence interval, *DALYs* disability-adjusted life years, *Diff* difference, *NS* neurological sequelae, *YLLs* years of life lost

* p-value < 0.05

^a^ The annual burden of malaria is estimated among all children < 5 in Uganda, including those that did not seek or receive medical treatment

^b^ This includes cases that sought care who may not have received treatment due to stock out of anti-malarials

^c^ Estimates the difference compared to the annual burden of malaria among cases that sought treatment

^d^ The impact of substandard and falsified anti-malarials is already incorporated in the annual burden of malaria. This impact could be reduced by improving the quality of medicines so all anti-malarials have an API > 85%

^e^ This scenario demonstrates the additional potential impact of antimicrobial resistance if treatment effectiveness of ACTs and quinine were reduced to the level of other treatments

SF anti-malarials contribute significantly to these estimates. Comparing the baseline among cases seeking treatment to a scenario with no SF drugs, poor quality anti-malarials were responsible for nearly 14,000 (8%) additional hospitalizations, approximately 300 (5%) more cases of neurological sequelae, and around 1100 (9%) additional deaths. SF anti-malarials contributed to about 71,000 (7%) years of potential life lost to children under five and almost 79,000 (7%) additional DALYs lost annually. In addition to the significant health impact, the economic impact of poor quality anti-malarials taken by Ugandan children was estimated at $31 million (8%). The additional morbidity and mortality caused by SF anti-malarials resulted in $5.2 million in direct costs annually, including $2.9 million incurred by the government and $2.3 million paid by patients as out-of-pocket costs. Productivity losses as a result of poor quality anti-malarials amounted to $25.9 million a year, including $8.1 million in caregiver productivity losses, $2 million due to disability, and $15.8 million due to early death.

In the event of widespread artemisinin resistance in Uganda, significant increases in severe malaria cases and hospitalizations would be expected. If treatment efficacies of ACT medicines and quinine were to fall to the level of other treatment efficacies, the model simulated a 6% increase in hospitalizations and 7% increase in malaria deaths, which would result in nearly 57,000 years of life lost due to early death and 63,000 DALYs lost. The resulting increase in economic impact was estimated at $48.5 million, a 12% increase in costs associated with paediatric malaria annually. This included an additional $8 million in government facility costs (25% increase) and an additional $7.5 million paid out-of-pocket by patients (30% increase).

## Discussion

The SAFARI model can be used to develop country-specific estimates of the health and economic impact of SF anti-malarials. Using agent-based modelling, we developed a dynamic model to simulate a cohort of children with demographic characteristics, malaria infection rates, care-seeking patterns, disease progression, treatment outcomes, and associated costs. Model results reinforce the magnitude of the burden of malaria at a national level, as well as highlight the health and economic benefits of reducing this burden through access to better quality medications. The SAFARI model should be used across malaria endemic countries to not only estimate the impact of SF anti-malarials, but to also assess the impact of national policies and interventions to counter this threat.

Through a case study in Uganda, this study demonstrated that SF anti-malarials contribute significantly to both the health and economic burden of malaria in children under five. The results of this model are useful as they provide the first country-specific estimate of the contribution of SF anti-malarials to the malaria burden for Uganda. It also demonstrates the burden of avertable costs on patients and the government. The results demonstrate that addressing drug quality, even with no changes in incidence rates, will significantly contribute to reducing the malaria burden. As such, these estimates are crucial to not only illustrate the scope of the issue to pertinent actors in the fight against malaria, but to also encourage and inform future research, policies, and interventions to combat poor quality anti-malarials.

The SAFARI model builds on and improves upon previous efforts to model the impact of malaria in sub-Saharan Africa [19, 23, 71–73]. For example, the disease model and clinical inputs utilized in the SAFARI model are comparable to previous efforts to model the burden of malaria in sub-Saharan Africa [19, 23, 71, 73]. Specifically, similar data inputs were utilized as Renshler et al. [23] estimating the number of under five deaths due to poor quality anti-malarials, Lubell et al. [71] modelling the impact of artemisinin resistance, and Bath et al. [19] assessing the economic impact of poor quality anti-malarials across sub-Saharan Africa. The SAFARI model builds upon the analysis by Bath et al. by focusing on a country, reducing the number of assumptions and better accounting for regional variation and heterogeneity within the model population. The SAFARI model is a country-level model and utilizes agent-based modelling rather than decision tree modelling, which can account for national, regional, and patient-specific variation, thereby generating more rigorous estimates. The SAFARI model also incorporates the financing source for direct costs and report epidemiological and cost outputs for each individual child in the model. The model can subsequently be used to assess various interventions to address the burden of malaria as well as assess the distribution of the malaria burden and impact of poor quality anti-malarials across demographic characteristics of a population.

The number of cases and deaths reported by the SAFARI model are comparable to those reported by the World Malaria Report [2]. While the World Malaria Report estimates the malaria burden across all ages, the WHO estimates that 45% of malaria cases occur in children under five [19]. This model reported approximately 3.5 million malaria cases, which is comparable to 45% of the estimated malaria cases in Uganda (2.0–5.6 million) reported by the 2017 World Malaria Report [2]. Children under five also account for the majority of malaria deaths; the nearly 29,000 total number of deaths resulting from the model is within the UNICEF estimates of 23,023–32,371 deaths due to malaria in 2016, calculated from the under-five mortality rate of 43.6–61.3 per 1000 live births with 6.7% of deaths due to malaria [74, 75]. Of children who sought care, the nearly 13,000 deaths reported by the model are in range of the 10,280–13,850 malaria treatment deaths reported in Uganda by the 2017 World Malaria Report [2]. Further, this study’s finding that 8.7% of under five deaths are due to SF anti-malarials is comparable to the estimated 3.8–8.9% of all-age malaria deaths due to poor quality medicines in sub-Saharan Africa reported by the WHO [19].

There are a number of key limitations to note. First, models are inherently limited by the quality of the data used to derive model parameters [76, 77]. To address this, an extensive literature search and MIS dataset analyses were conducted to ensure that model parameters were derived from the most recent and best quality published data. While the quality of data inputs inherently limit model outputs, the SAFARI model improves upon previous estimates by appropriately controlling for these limitations through rigorous analyses. Secondly, population, regional, and individual level heterogeneity limits models to capture population-wide variances [76, 77]. To control for heterogeneity, a probabilistic agent-based model was used and each agent-child was assigned with demographic characteristics including age, sex, geographic region, rural/urban, wealth quintile, and level of maternal education. Using these demographic characteristics, the SAFARI model is able to account for natural individual and regional heterogeneity based on the variance around each input variable and assign individual rates specific to each agent-child’s characteristic and location. Third, a large amount of uncertainty in the case fatality rate of untreated malaria cases was found, with estimates ranging from 0.45 to 60% in the literature [19, 23, 50, 60]. Due to this uncertainty, we calibrated the model using various literature source estimates for this rate. Further, the model does not account for malaria treatments taken by children that do not have malaria and unessential inpatient care for non-severe cases due to lack of data. Despite these limitations, the SAFARI model is a unique and valuable tool to assess and counter the impact of SF anti-malarials at the country level.

## Conclusions

After years of progress in addressing the burden of malaria, recent indicators suggest that malaria is making a resurgence. To ensure that progress continues, it is essential to address the factors affecting this resurgence, such as the rise in poor quality anti-malarials. The SAFARI model is presented to assess and illustrate the magnitude of the health and economic impact of SF anti-malarials at the country level through a model case study in Uganda. The SAFARI model should inform country malaria stakeholders, international donors, and national malaria control programmes to recognize the burden of SF anti-malarials and identify interventions to improve medicinal quality. As the world seeks to achieve the Sustainable Development Goals and meet the Global Health Security Agenda, reducing the prevalence of poor quality medicines will be essential to combat the global burden of malaria.

## Additional file

**Additional file 1.** Additional model inputs and coefficients.

## Abbreviations

ACT: artemisinin-based combination therapy
API: active pharmaceutical ingredient
AQUAMAT: African Quinine Artesunate Malaria Treatment Trial
DALYs: disability-adjusted life years
GDP: gross domestic product
MIS: Malaria Indicator Survey
SAFARI: Substandard and Falsified Antimalarial Research Impact model
UNICEF: United Nations International Children’s Emergency Fund
WHO: World Health Organization
WWARN: Worldwide Antimalarial Resistance Network
