# Supplementary material for: Development of an agent-based model to assess the impact of substandard and falsified anti-malarials: Uganda case study
Source: Malar J. 2019 Jan 9;18:5. doi: 10.1186/s12936-018-2628-3 (PMC6327614; doi:10.1186/s12936-018-2628-3)
Supplement: Supplementary file 1 — Additional file 1. Additional model inputs and coefficients. [file 12936_2018_2628_MOESM1_ESM.docx]

**Development of an agent-based model to assess the impact of substandard and falsified antimalarials: Uganda case study**

Appendix 1. Demographic and Health Data from the 2016 Uganda Malaria Indicator Survey^1,2^

| **Demographics** | **Proportions** |
| --- | --- |
| Children in Region 1 | 0.121 |
| Children in Region 2 | 0.101 |
| Children in Region 3 | 0.120 |
| Children in Region 4 | 0.041 |
| Children in Region 5 | 0.108 |
| Children in Region 6 | 0.119 |
| Children in Region 7 | 0.104 |
| Children in Region 8 | 0.101 |
| Children in Region 9 | 0.108 |
| Children in Region 10 | 0.078 |
| Urban Children in Region 1 | 0.255 |
| Urban Children in Region 2 | 0.206 |
| Urban Children in Region 3 | 0.119 |
| Urban Children in Region 4 | 1.000 |
| Urban Children in Region 5 | 0.093 |
| Urban Children in Region 6 | 0.109 |
| Urban Children in Region 7 | 0.102 |
| Urban Children in Region 8 | 0.043 |
| Urban Children in Region 9 | 0.147 |
| Urban Children in Region 10 | 0.123 |
| Urban Children in Lowest SES | 0.061 |
| Urban Children in Low SES | 0.028 |
| Urban Children in Middle SES | 0.065 |
| Urban Children in High SES | 0.169 |
| Urban Children in Highest SES | 0.677 |
| Rural Children in Lowest SES | 0.229 |
| Rural Children in Low SES | 0.235 |
| Rural Children in Middle SES | 0.228 |
| Rural Children in High SES | 0.206 |
| Rural Children in Highest SES | 0.102 |
| Lowest SES Children with Mother's with No Education | 0.325 |
| Low SES Children with Mother's with No Education | 0.194 |
| Middle SES Children with Mother's with No Education | 0.161 |
| High SES Children with Mother's with No Education | 0.149 |
| Highest SES Children with Mother's with No Education | 0.023 |
| Lowest SES Children with Mother's with a Primary Education | 0.629 |
| Low SES Children with Mother's with a Primary Education | 0.701 |
| Middle SES Children with Mother's with a Primary Education | 0.682 |
| High SES Children with Mother's with a Primary Education | 0.597 |
| Highest SES Children with Mother's with a Primary Education | 0.382 |
| Lowest SES Children with Mother's with a Secondary Education | 0.046 |
| Low SES Children with Mother's with a Secondary Education | 0.106 |
| Middle SES Children with Mother's with a Secondary Education | 0.158 |
| High SES Children with Mother's with a Secondary Education | 0.254 |
| Highest SES Children with Mother's with a Secondary Education | 0.595 |
|  |  |
| **Malaria Prevalence** | **Coefficients** |
| Region 1 | 0.551 |
| Region 2 | 1.245 |
| Region 3 | 1.925 |
| Region 4 | 0.020 |
| Region 5 | 1.030 |
| Region 6 | 0.923 |
| Region 7 | 0.715 |
| Region 8 | 1.443 |
| Region 9 | 0.217 |
| Region 10 | 1.452 |
| Urban | 0.335 |
| Rural | 1.127 |
| SES Quintile 1 | 1.474 |
| SES Quintile 2 | 1.212 |
| SES Quintile 3 | 1.193 |
| SES Quintile 4 | 0.720 |
| SES Quintile 5 | 0.206 |
| Maternal Education (None) | 1.500 |
| Maternal Education (Primary) | 1.039 |
| Maternal Education (Secondary+) | 0.462 |
| Age Category 1 (<6 Months) | 0.414 |
| Age Category 2 | 0.000 |
| Age Category 3 | 0.000 |
| Age Category 4 | 0.671 |
| Age Category 5 | 0.887 |
| Age Category 6 | 1.166 |
| Age Category 7 | 1.129 |
| Age Category 8 | 1.370 |
|  |  |
| **Health Care Seeking** | **Coefficients** |
| *Public facilities* |  |
| SES Quintile 1 | 1.068 |
| SES Quintile 2 | 1.020 |
| SES Quintile 3 | 1.229 |
| SES Quintile 4 | 0.908 |
| SES Quintile 5 | 0.580 |
| Maternal Education (None) | 0.977 |
| Maternal Education (Primary) | 1.085 |
| Maternal Education (Secondary+) | 0.736 |
| Age Category 1 (<6 Months) | 1.037 |
| Age Category 2 | 1.224 |
| Age Category 3 | 0.803 |
| Age Category 4 | 0.993 |
| Age Category 5 | 0.951 |
| Age Category 6 | 1.004 |
| Age Category 7 | 0.970 |
| Age Category 8 | 1.067 |
| Urban | 0.921 |
| Rural | 1.012 |
| *Private facilities* |  |
| SES Quintile 1 | 0.763 |
| SES Quintile 2 | 0.971 |
| SES Quintile 3 | 0.883 |
| SES Quintile 4 | 1.168 |
| SES Quintile 5 | 1.576 |
| Maternal Education (None) | 0.943 |
| Maternal Education (Primary) | 0.880 |
| Maternal Education (Secondary+) | 1.469 |
| Age Category 1 (<6 Months) | 0.866 |
| Age Category 2 | 1.203 |
| Age Category 3 | 1.240 |
| Age Category 4 | 1.074 |
| Age Category 5 | 1.041 |
| Age Category 6 | 1.012 |
| Age Category 7 | 1.010 |
| Age Category 8 | 0.739 |
| Urban | 1.195 |
| Rural | 0.971 |
| *Pharmacies* |  |
| SES Quintile 1 | 0.000 |
| SES Quintile 2 | 0.578 |
| SES Quintile 3 | 0.000 |
| SES Quintile 4 | 1.745 |
| SES Quintile 5 | 4.699 |
| Maternal Education (None) | 0.000 |
| Maternal Education (Primary) | 0.972 |
| Maternal Education (Secondary+) | 2.183 |
| Age Category 1 (<6 Months) | 0.651 |
| Age Category 2 | 3.215 |
| Age Category 3 | 1.052 |
| Age Category 4 | 0.198 |
| Age Category 5 | 2.245 |
| Age Category 6 | 0.849 |
| Age Category 7 | 0.700 |
| Age Category 8 | 0.689 |
| Urban | 4.546 |
| Rural | 0.471 |
| *Drug Stores* |  |
| SES Quintile 1 | 1.213 |
| SES Quintile 2 | 1.066 |
| SES Quintile 3 | 1.042 |
| SES Quintile 4 | 0.740 |
| SES Quintile 5 | 0.634 |
| Maternal Education (None) | 1.526 |
| Maternal Education (Primary) | 1.034 |
| Maternal Education (Secondary+) | 0.314 |
| Age Category 1 (<6 Months) | 1.145 |
| Age Category 2 | 0.550 |
| Age Category 3 | 1.110 |
| Age Category 4 | 0.705 |
| Age Category 5 | 0.727 |
| Age Category 6 | 1.096 |
| Age Category 7 | 1.136 |
| Age Category 8 | 1.252 |
| Urban | 0.696 |
| Rural | 1.045 |
| *CHWs* |  |
| SES Quintile 1 | 2.128 |
| SES Quintile 2 | 0.000 |
| SES Quintile 3 | 1.399 |
| SES Quintile 4 | 0.725 |
| SES Quintile 5 | 0.000 |
| Maternal Education (None) | 1.811 |
| Maternal Education (Primary) | 0.852 |
| Maternal Education (Secondary+) | 0.618 |
| Age Category 1 (<6 Months) | 0.279 |
| Age Category 2 | 0.000 |
| Age Category 3 | 3.321 |
| Age Category 4 | 0.000 |
| Age Category 5 | 0.782 |
| Age Category 6 | 1.816 |
| Age Category 7 | 1.125 |
| Age Category 8 | 0.471 |
| Urban | 1.394 |
| Rural | 0.941 |
| *Self-treat/neighbours* |  |
| SES Quintile 1 | 1.318 |
| SES Quintile 2 | 1.054 |
| SES Quintile 3 | 0.886 |
| SES Quintile 4 | 0.965 |
| SES Quintile 5 | 0.362 |
| Maternal Education (None) | 0.856 |
| Maternal Education (Primary) | 1.162 |
| Maternal Education (Secondary+) | 0.609 |
| Age Category 1 (<6 Months) | 1.085 |
| Age Category 2 | 0.146 |
| Age Category 3 | 0.767 |
| Age Category 4 | 1.082 |
| Age Category 5 | 0.731 |
| Age Category 6 | 0.857 |
| Age Category 7 | 1.164 |
| Age Category 8 | 1.517 |
| Urban | 0.460 |
| Rural | 1.081 |
| *No Treatment* |  |
| SES Quintile 1 | 1.525 |
| SES Quintile 2 | 1.139 |
| SES Quintile 3 | 0.714 |
| SES Quintile 4 | 0.469 |
| SES Quintile 5 | 0.580 |
| Maternal Education (None) | 1.551 |
| Maternal Education (Primary) | 0.964 |
| Maternal Education (Secondary+) | 0.522 |
| Age Category 1 (<6 Months) | 1.726 |
| Age Category 2 | 0.053 |
| Age Category 3 | 0.461 |
| Age Category 4 | 0.867 |
| Age Category 5 | 1.881 |
| Age Category 6 | 1.054 |
| Age Category 7 | 0.542 |
| Age Category 8 | 1.234 |
| Urban | 0.862 |
| Rural | 1.021 |
|  |  |
| **Severe Malaria** | **Coefficients** |
| SES Quintile 1 | 0.721 |
| SES Quintile 2 | 1.262 |
| SES Quintile 3 | 1.275 |
| SES Quintile 4 | 1.008 |
| SES Quintile 5 | 0.402 |
| Maternal Education (None) | 1.078 |
| Maternal Education (Primary) | 0.958 |
| Maternal Education (Secondary+) | 1.063 |
| Age Category 1 (<6 Months) | 0.000 |
| Age Category 2 | 0.387 |
| Age Category 3 | 1.867 |
| Age Category 4 | 1.487 |
| Age Category 5 | 2.144 |
| Age Category 6 | 0.898 |
| Age Category 7 | 0.786 |
| Age Category 8 | 0.740 |
| Urban | 1.761 |
| Rural | 0.957 |

CHWs: Community Health Workers; SES: Socio-Economic Status.

References

1. Uganda Bureau of Statistics [Uganda], National Malaria Control Programme [Uganda], Uganda Malaria Surveilance Project Molecular Laboratory [Uganda], and ICF. 2018. Uganda Demographic and Health Survey 2016 [Dataset]. UGKR72.DTA. Rockville, Maryland: Uganda Bureau of Statistics, National Malaria Control Programme, Uganda Malaria Surveillance Project Molecular Laboratory, and ICF [Producers]. ICF [Distributor], 2015.
2. Uganda Bureau of Statistics [Uganda], National Malaria Control Programme [Uganda], Uganda Malaria Surveillance Project Molecular Laboratory [Uganda], and ICF. 2018. Uganda Demographic and Health Survey 2016 [Dataset]. UGPR72.DTA. Rockville, Maryland: Uganda Bureau of Statistics, National Malaria Control Programme, Uganda Malaria Surveillance Project Molecular Laboratory, and ICF [Producers]. ICF [Distributor], 2015.
